# Preoperative antisepsis in ophthalmic surgery (a review)

**DOI:** 10.22336/rjo.2021.25

**Published:** 2021

**Authors:** Alexandra-Cătălina Zaharia, Otilia-Maria Dumitrescu, Roxana-Elena Rogoz, Andreea Elena Dimirache, Mihail Zemba

**Affiliations:** * Ophthalmology Department, “Dr. Carol Davila” Central Military Emergency Hospital, Bucharest, Romania

**Keywords:** endophthalmitis, antisepsis, ophthalmic surgery, preoperative preparation, povidone-iodine

## Abstract

Endophthalmitis remains a serious complication following intraocular procedures. Preoperative prophylactic measures for endophthalmitis decrease the morbidity associated with this disease and represent a standard of care prior to ophthalmic surgery. The literature supports as measures for ocular antisepsis: povidone-iodine solution for ocular surface preparation, chlorhexidine in patients with iodine allergy and application of topical antibiotics. Povidone-iodine is regarded as the most effective antiseptic associated with significant reduction in ocular surface bacterial counts. Currently, the recommended preoperative management is the application of 5% povidone-iodine solution in the conjunctival fornix, prior to surgery. This paper reviews the preoperative measures for ocular antisepsis, used in order to decrease the risk of culture-proven endophthalmitis.

## Introduction

Infectious endophthalmitis is a potentially devastating complication following intraocular procedures, being attributable to bacterial or fungal infection. Most causative organisms in acute postoperative endophthalmitis come from the patient’s own periocular flora. A major objective of preoperative and intraoperative management is the prevention of endophthalmitis by reducing the pathogenic organism entry in the anterior chamber. 

The ocular surface of healthy individuals is normally colonized by aerobic and anaerobic bacteria that form the commensal (normal) bacterial flora of the conjunctiva. The most common bacteria identified on the ocular surface are coagulase-negative staphylococci (Staphylococcus epidermidis > 60%), diphtheroids and Propionibacterium acnes. Studies of conjunctival flora showed that the spectrum of bacteria varies among major age groups, such that children tend to have more Streptococcus species [**1**]. 

There are a few methods of decreasing the infection rate preoperatively. The literature supports povidone-iodine (PI) use as the most important method of preoperative antisepsis, prior to ocular surgeries. In patients with iodine allergy, 0.05% aqueous chlorhexidine may be used. This measure can pe supplemented with preoperative topical antibiotics, although none of the studies has demonstrated their actual efficacy in lowering the risk of endophthalmitis when they are administered prior to surgery.

## Normal ocular flora, causing postoperative endophthalmitis

The conjunctiva has many defensive mechanisms that operate in synergy, preventing and limiting infections. The normal conjunctival flora harbors several aerobic and anaerobic bacteria that maintain surface homeostasis and immunoregulation. Studies of the conjunctival flora suggest that the most common bacteria cultured from the ocular surface is Staphylococcus epidermidis. Techniques of molecular cloning and DNA sequencing helped identify a great diversity of conjunctival bacteria, including Corynebacterium, Propionibacterium, Klebsiella species. The conjunctival flora in children has less bacterial species and tends to have more Streptococcus species [**2**]. 

The most commonly isolated microbial species in postoperative endophthalmitis are Gram-positive bacteria (Staphylococcus epidermidis 33-77%, Streptococcus viridans, and Staphylococcus aureus 10-21%) [**3**]. Sensitive genetic testing demonstrated that in Staphylococcus epidermitis endophthalmitis, the causative organism comes from the patient’s own periocular flora and enters the eye by direct inoculation during surgery [**4**]. Moreover, studies revealed that the microbial spectrum of postoperative endophthalmitis varies in different parts of the world. For example, in the USA, coagulase-negative staphylococci are the most common isolates from postoperative endophthalmitis following cataract surgery. Endophthalmitis following intravitreal injection is more commonly associated with more virulent organisms such as streptococci [**5**]. 

Fungal colonization of the ocular surface is less common, having a prevalence of up to 8%. Most fungal post-cataract endophthalmitis are due to filamentous fungi (Aspergillus species) [**3**]. 

Therefore, these ideas emphasize the importance of the patient’s own microbial flora in determining the etiology of exogenous endophthalmitis. Because the periocular skin and lashes are possible sources of endophthalmitis, careful preoperative clinical evaluation of the patient should not be neglected as it is an essential prevention measure. Clinicians should look for evidence of periocular infection, as these conditions should be treated before intraocular surgery is performed. Careful ophthalmic examination may reveal signs of periocular cutaneous disease, staphylococcal or seborrheic blepharitis, conjunctival hyperemia, hordeolum, chalazion, dacryocystitis, history of prolonged use of corticosteroids, and other regional infections. When performing surgical draping, an important measure is the complete covering of the eyelashes to minimize contamination of the wound with eyelid flora.

## Preoperative antisepsis

**A. Povidone-iodine**

Preoperative antisepsis with topical povidone-iodine (PI) has become a standard of care for endophthalmitis prophylaxis. By the 1980s, studies supported the essential role of PI for application on the ocular surface. Apt et al. evidenced a reduction of bacterial colonies and species on the conjunctiva after using a half strength povidone-iodine solution topically, as part of the preoperative chemical preparation of the eye [**6**]. 

PI is an antimicrobial agent, routinely used as a surgical scrub for skin disinfection. Povidone-iodine is a complex of iodide and polyvinylpyrrolidone that penetrates the cytoplasmic membrane, being bactericidal against drug-resistant bacteria. The effect is attributable to the release of free iodine to the target cell surface by povidone, which acts as a carrier. PI has broad-spectrum antimicrobial activity, with efficacy against bacteria, fungi, protozoa and viruses [**7**].

Prophylaxis of endophthalmitis with topical povidone-iodine was evaluated in a single-center randomized study between January 1988 and February 1990, conducted at the New York Eye and Ear Infirmary of Mount Sinai, New York, USA. The preoperative conjunctival preparation was made with two different antiseptics: Argyrol, a solution of mild silver protein at varying strengths and 5% povidone-iodine. There was a statistically significant reduction of endophthalmitis with preoperative conjunctival application of 5% PI [**5**].

The ideal concentration of PI for maximal efficacy has not been established and studies have examined the use of different PI concentrations with varying results. Dilute PI is described as a concentration of 1% or less, while 5%, 10% PI is considered a concentrated solution. In 1982, Berkelman et al. demonstrated that dilute PI solution (0.1%-1%) has greater bactericidal activity than a full-strength solution (10%) and kills bacteria at least as quickly as higher concentrations [**8**]. The contact killing time of PI varies from 10 to 900 seconds (15 min), but most of the microbes are killed in less than 60 seconds [**5**]. PI with higher concentrations may take longer to kill microorganisms due to the lower free iodine concentration [**7**]. 

Many studies approve the idea that 5% PI effectively decreases the bacterial flora of the ocular surface and directly influences the reduction in the incidence of endophthalmitis after cataract surgery. This concentration is preferred due to the evidence base that supports its use and due to concerns over the toxicity of the undiluted form. Ferguson et al. concluded in a study from 2002 that 5% PI is more effective than 1% PI in decreasing the human conjunctival bacterial load, showing a more increased bactericidal activity of 5% PI, thus supporting its use rather than 1% PI. The study found that 5% PI was more efficient than 1% PI in the presence of heavier initial bacterial load. Although previous studies considered 1% PI more bactericidal, it has a lower reservoir of available iodine, which is depleted when the bacterial load is increased [**9**]. Halachimi et al. evaluated the effectiveness of PI 5% alone compared to adding topical moxifloxacin 0.5% to topical 5% PI, in the preoperative reduction of bacterial recovery from the conjunctiva, in 464 patients. The study concluded that adding moxifloxacin had no significant effect, highlighting the benefits of using 5% PI alone [**10**]. 

Wu et al. studied the ideal concentration of PI for ophthalmic disinfection, prior to extracapsular cataract extraction with intraocular lens implantation, assessing the risk of postoperative endophthalmitis associated with different prophylactic protocols, over an 8-year period. They demonstrated that preoperative skin disinfection with 10% PI and conjunctival disinfection with 5% PI significantly reduced the relative risk of postoperative endophthalmitis. Furthermore, it was showed that PI should dry after skin preparation and the conjunctival fornices should not be irrigated before 1 minute contact with the PI solution [**11**].

The European Society of Cataract and Refractive Surgeons recommends applying povidone-iodine 5%-10% to the cornea, conjunctival sac, and periocular skin for a duration of minimum 3 minutes prior to surgery. Carrim et al. analyzed this recommendation on 54 patients undergoing unilateral cataract surgery by phacoemulsification and their results supported the significant reduction in organisms such as Coagulase-negative Staphylococcus from the lid and the conjunctival flora [**12**]. 

Ciulla et al. gathered a substantial body of literature for a systematic review to assess commonly used cataract surgery bacterial endophthalmitis prophylaxis techniques. The recommendations were ranked into two categories: clinical rating (level A, B, C) and evidence rating (level I, II, III). Preoperative PI antisepsis was the only prophylactic measure that had superior rating for prevention of post cataract surgery endophthalmitis, with level B on the clinical rating category and level II on the evidence rating. The other prophylactic measures evaluated were: subconjunctival antibiotics, preoperative lash trimming, saline-irrigation, topical antibiotics and the use of irrigating solutions containing antibiotics, which received the level C on the clinical rating and level III on the evidence rating [**13**]. 

Yanai et al. studied the cytotoxicity of PI on human corneal epithelial cells, which was reduced compared to that of the other agents used. Concentrations as low as 0.0125% produced corneal epithelial toxicity in human cultured cells and the toxicity increased with PI concentration and exposure time [**7**,**14**]. Another study that examined microbial decontamination of human eyes donated for transplantation demonstrated that 5% PI applied for 2 minutes is sufficient for maximal decontamination without the risk of iodine penetrating the corneal stromal layers and damaging corneal fibroblasts. The study found that iodine can penetrate the corneal tissue from the epithelium and half way in the stroma, depending on the depth of concentration and exposure time [**15**]. Other documented toxic effects of topical PI include conjunctival irritation, punctate epitheliopathy and less common, contact dermatitis [**9**]. 

A study in 1981 revealed the contamination of PI solution with Pseudomonas cepacia, after identifying the bacteria in the blood cultures of 52 patients in four hospitals, over a 7-month period. Possible hypotheses to explain the survival of the bacteria in the PI solutions were analyzed. First, it was thought that the PI solution contained a low concentration of available iodine, but the level of free iodine, the component that possesses the microbicidal activity, was similar with the other PI solutions analyzed. The best supported hypothesis was that the isolated strains of Pseudomonas cepacia had a greater tolerance to free iodine found in the PI solution. It was found that the blood cultures were more likely to be positive for the bacteria when the PI solution was left longer on the skin. These observations emphasized the safety need to prepare fresh PI solution every day, discarding the remaining solution, in order to prevent an undesirable contamination [**5**,**16**].

**B. Chlorhexidine**

Even though PI has been long established as the gold standard for endophthalmitis prophylaxis prior to intraocular procedures, some patients experience iodine sensitivity or allergy. Chlorhexidine is a topical antiseptic that has been used in the healthcare field since 1954. It is a cationic biguanide that damages the bacterial structure causing a disorganization of the cytoplasmic membrane and subsequent cell death. 

Chlorhexidine has broad-spectrum activity against gram positive and gram-negative bacteria, fungi, and some lipid-enveloped viruses, but is not effective against spores.

The ideal concentration and contact time for chlorhexidine application is yet to be determined. Chlorhexidine can be used in concentrations ranging from 0.02% to 4%. For preoperative antisepsis, 0.05% concentration is well tolerated.

However, there are safety concerns on the use of chlorhexidine, because it has not been investigated adequately as prophylaxis for postoperative endophthalmitis [**17**]. 

**C. Antibiotic Prophylaxis**

The use of PI in the moments just prior to the beginning of the actual surgery, both during and after draping the eye, has proven to be the most, if not the only, effective method of preventing endophthalmitis after intraocular surgery. However, additional means of reducing the incidence of this dramatic complication have been studied. Antibiotics have long been used not only as curative, but also as prophylactic agents, and have been administered preoperatively, during surgery, as well as postoperatively. Antibiotic administration routes include topical, intracameral, subconjunctival, subtenon, retrobulbar or peribulbar injections and via the balanced saline irrigating solution (BSS). There are multiple studies that have attempted to establish the usefulness of preoperative antibiotic use in the prevention of postoperative infectious complications [**18**]. 

Among the first such studies was the one by Christie and colleagues, who advocated that the use of penicillin injected periocularly would substantially reduce the risk of developing endophthalmitis after cataract surgery [**19**]. 

A study by Sobaci at al. reported a reduced contamination of aqueous humor with the use of vancomycin and gentamicin in the irrigating solution. In the same study, the only two cases of endophthalmitis after cataract surgery occurred in the control group, in which only BSS was used as irrigation. However, both cases were also associated with intraoperative complications (rupture of the posterior capsule) and, as such, it is difficult to assess the causative relationship between this complication and the lack of antibiotic in the irrigating solution [**20**]. 

In the European Society of Cataract and Refractive Surgeons (ESCRS) 2007, a multicenter randomized controlled study including 16,211 patients that investigated the prophylactic means for endophthalmitis following cataract surgery, the use of intracameral cefuroxime injections at the end of the surgery resulted in a significant 5-fold reduction in the incidence of endophthalmitis evaluated at six weeks after surgery. This occurred independently from the concurrent administration of topical levofloxacin. The administration of intracameral cefuroxime did not appear to affect the final visual acuity in the cases of those who developed endophthalmitis [**3**]. Similarly, an Indian study of 617,453 eyes undergoing cataract surgery found a 3.5-fold overall reduction in the rate of postoperative endophthalmitis with the use of intracameral moxifloxacin at the conclusion of surgery [**21**].

Nevertheless, there are also studies which reported that the use of antibiotic prophylaxis is detrimental. Thus, two studies that investigated the rate of endophthalmitis after intravitreal injection of anti-VEGF agents showed a 1.7 and, respectively, a 3 times greater risk of developing endophthalmitis if antibiotics are used [**22**,**23**]. Possible explanations include the overall detrimental effect on the homeostasis and health of the ocular surface and, also, the selection of bacteria with multiple resistance profiles [**23**]. Indeed, another study explored the resistance profiles of agents implicated in acute postoperative endophthalmitis after cataract surgery over a period of 10 years and found an increase in the MRSA infections and in the bacterial resistance to fluoroquinolones (ciprofloxacin and moxifloxacin) and to oxacillin [**24**]. 

Overall, George and Steward [**24**] concluded that the use of antibiotic prophylaxis should not replace the thorough preparation of the operating room and sterile techniques, as, unless strict antisepsis is maintained, the risk of endophthalmitis will always be high, despite the use of antibiotics. 

## Conclusions

Complete sterilization of the ocular surface prior to intraocular surgery should not be expected, because no antiseptic regimen was found to consistently decontaminate the ocular surface throughout the perioperative period. PI solution has been studied extensively and is strongly supported as the most efficient topical antiseptic that reduces the risk of postoperative endophthalmitis. Currently, ophthalmic preparation with 5% dilute PI prior to surgery has become a standard of care. Aqueous chlorhexidine 0.05% may be used when there is a contraindication in using PI. While preoperative antisepsis with PI or chlorhexidine is mandatory to reduce the conjunctival bacterial load, the value of preoperative topical antibiotic therapy is uncertain.

**Conflict of Interest statement**

Authors state no conflict of interest.

**Acknowledgements**

None.

**Sources of Funding**

None.

**Disclosures**

None.
